# Target Organ Damage in Hypertension

**DOI:** 10.1155/2012/454508

**Published:** 2012-07-05

**Authors:** Mario Fritsch Neves, Agostino Virdis, Wille Oigman

**Affiliations:** ^1^Department of Clinical Medicine, State University of Rio de Janeiro, 20551030 Rio de Janeiro, RJ, Brazil; ^2^Department of Internal Medicine, University of Pisa, 56100 Pisa, Italy

This special issue is related to the main concern in hypertensive disease: the target-organ damage. The selected papers approach some aspects of renal and cerebral disease associated to hypertension but the principal focus is on the arterial vessel as the main organ involved in hypertensive patients. Although hypertensive cardiopathy is not specifically included in this issue, the heart disease might be considered as one of the principal consequences of vascular dysfunction discussed in many papers.

Cardiovascular disease has been associated to a low level of chronic inflammatory state which may be an important mechanism leading to organ damage in hypertensive patients. Interestingly, the paper by D. A. B. Kasal and E. L. Schiffrin provides new evidences about the role of the T-regulatory lymphocytes, showing that this population of lymphocytes may inhibit the inflammatory process resulting in beneficial effects on vascular disease in hypertension. This review also establishes the linkage between the immune response, renin angiotensin aldosterone system, and oxidative stress. Recently, the same authors published original papers reporting that T-regulatory lymphocytes were able to prevent vascular injury mediated by angiotensin II and aldosterone by suppressing inflammation and oxidative stress and improving endothelial function in experimental models of hypertension [1, 2].

Vascular disease is the main topic in three review papers of this collection. In the first review, methods to evaluate endothelial dysfunction were considered in experimental models of hypertension. It is important to recognize that a large range of knowledge of changes in microcirculation came from vascular studies using resistance arteries mounted in wire or pressurized myograph in different animal models of hypertension and more recently in clinical settings of hypertensive disease. In the second review, A. R. Cunha et al. describe the controversial role of magnesium in the pathogenesis and vascular complications of hypertension. In spite of heterogeneity of study populations, the authors suggest that magnesium is more involved in the functional vascular changes and also on local metabolic stability with no influence on the vascular structure. Accordingly, experimental and epidemiological studies reported a connection between intracellular concentrations of ions and development of hypertension and other cardiovascular diseases [3, 4]. In the third review, V. Javaroni and M. Fritsch raise the question about the connection between erectile dysfunction and hypertension. Despite the high prevalence of sexual dysfunction among hypertensive men, unfortunately erectile dysfunction is usually not yet considered among subjects with increased blood pressure, neither in the initial nor even in the follow-up evaluation. In fact, erectile dysfunction may be the first manifestation of endothelial dysfunction in hypertensive men, and for this reason it can be indicated as a risk marker of cardiovascular events [5, 6]. Endothelial dysfunction is the main mechanism linking both conditions and seems to be correlated to effectiveness of phosphodiesterase-5 inhibitors [7].

Vascular stiffness was the concern of the original study carried out by I. Farro et al. in Uruguayan subjects. The main objective of that study was to discriminate the reference values of pulse wave velocity in a Uruguayan population. To our knowledge, this is the first study that evaluates normal values of pulse wave velocity in this population, which may help to characterize the vascular aging in these individuals. Unlike the pulse wave velocity determination, which has been used more in research area, carotid intima-media thickness (IMT) and ankle-brachial index (ABI) measurements have been more commonly obtained since they represent a relationship with atherosclerosis process. Indeed, both values may be used for cardiovascular risk stratification in hypertensive patients and therefore are included in recent guidelines of hypertension [8, 9]. Following this reasoning, M. Trindade et al. were able to identify pulse pressure, HDL-cholesterol, and C-reactive protein as variables associated to increased carotid IMT in treated hypertensive women with no history of diabetes or cardiovascular events. Since these results cannot be extrapolated to other hypertensive subjects, further studies are needed for a better identification of clinical, metabolic, and vascular parameters that can begin or accelerate atherosclerosis in hypertension. In other cross-sectional study, R. Monteiro et al. reported that diabetes, metabolic syndrome, increased pulse pressure, and high Framingham risk score were associated to low ABI in elderly hypertensive subjects. Interestingly, a simple modification of ABI calculation, using lower instead of higher ankle pressure, was able to identify more patients at high risk and include smoking and LDL-cholesterol as variables associated to low ABI in hypertensive patients over 65 years old.

The strong connection between hypertension and chronic kidney disease is clear when we recognize the kidney as an important source of changes involved in the pathogenesis of essential hypertension and at the same time a frequent target for organ damage in hypertensive patients. The original paper by R. A. Gismondi et al. addresses the relationship between kidney disease and vascular stiffness. In a case-control study, comparing to hypertensive subjects with normal renal function, hypertensive patients with low glomerular filtration rate (<60 mL/min) presented higher ambulatory arterial stiffness index which was correlated to pulse pressure, suggesting increased vascular stiffness in this population. And last but not least, the brain was the focus in the last review paper. F. Medeiros et al. provide evidences concerning the influence of dietary interventions that can reduce the risk of stroke in hypertension. The authors raise the controversial benefit of carotenoids, flavonoids, n-3 polyunsaturated fats, and lower salt and high glycemic index intake in risk of stroke. 

In summary, this special issue covers a wide range of target organ damage associated to hypertension, with a special attention to the vessel as the central key not only for vascular disease but also for cardiac, cerebral, and renal complications in hypertensive patients. Taken together, these papers point out the importance of a complete approach of each hypertensive subject beyond the mere blood pressure measurement.

*Mario Fritsch Neves* *Mario Fritsch Neves* *Agostino Virdis* *Agostino Virdis* *Wille Oigman* *Wille Oigman*
